# Isolation and Characterization of Lignin from Sapele (*Entandrophragma cylindricum*): Application in Flexible Polyurethane Foam Production

**DOI:** 10.3390/polym17152156

**Published:** 2025-08-06

**Authors:** Hubert Justin Nnanga Guissele, Arnaud Maxime Cheumani Yona, Armel Edwige Mewoli, Désiré Chimeni-Yomeni, Lucioni Fabien Tsague, Tatiane Marina Abo, Jean-Bosco Saha-Tchinda, Maurice Kor Ndikontar, Antonio Pizzi

**Affiliations:** 1 Laboratory of Macromolecular Chemistry, Department of Inorganic Chemistry, University of Yaoundé I, Yaoundé P.O. Box 812, Cameroon; acheumani@gmail.com (A.M.C.Y.); lucionitsague@gmail.com (L.F.T.); tatianemarinaa@yahoo.com (T.M.A.); saha_jb@yahoo.fr (J.-B.S.-T.); mndikontar@gmail.com (M.K.N.); 2 Laboratory of Engineering Civil and Mechanic, National Advanced School of Engineering, University of Yaoundé I, Yaoundé P.O. Box 812, Cameroon; mewoliarmel@yahoo.fr; 3 LABPLAS, 1951 Rue Nobel, Sainte-Julie, QC J3E 1Z6, Canada; dchimeniyomeni@labplas.com; 4 ENSTIB-LERMAB, University of Lorraine, 27 Rue Philippe Seguin, P.O. Box 21042, 88051 Epinal, Cedex 9, France

**Keywords:** lignin isolation, lignin liquefaction, hydroxyl number, bio-polyols, polyurethane foams

## Abstract

Lignin used in this work was isolated from sapele (*Entandrophragma cylindricum*) wood through a hybrid pulping process using soda/ethanol as pulping liquor and denoted soda-oxyethylated lignin (SOL). SOL was mixed with a polyethylene glycol (PEG)–glycerol mixture (80/20 *v*/*v*) as liquefying solvent with 98% wt. sulfur acid as catalyst, and the mixture was taken to boil at 140 °C for 2, 2.5, and 3 h. Three bio-polyols LBP1, LBP2, and LBP3 were obtained, and each of them exhibited a high proportion of -OH groups. Lignin-based polyurethane foams (LBPUFs) were prepared using the bio-polyols obtained with a toluene diisocyanate (TDI) prepolymer by the one-shot method. Gel permeation chromatography (GPC), Fourier transform infrared spectroscopy (FTIR), and carbon-13 nuclear magnetic resonance spectroscopy (^13^C NMR) were used characterize lignin in order to determine viscosity, yield, and composition and to characterize their structure. The PEG-400–glycerol mixture was found to react with the lignin bio-polyols’ phenolic -OHs. The bio-polyols’ viscosity was found to increase as the liquefaction temperature increased, while simultaneously their molecular weights decreased. All the NCO groups were eliminated from the samples, which had high thermal stability as the liquefaction temperature increased, leading to a decrease in cell size, density, and crystallinity and an improvement in mechanical performance. Based on these properties, especially the presence of some aromatic rings in the bio-polyols, the foams produced can be useful in automotive applications and for floor carpets.

## 1. Introduction

Lignin is known to be a renewable and low-cost resource. It is a by-product of lignocellulosic pulping commonly used as a combustible for heat production. Due to the -OH groups in its structure, it is used as an additive to polyol polymers or as a substitute for petrochemical products that are non-biodegradable and cause environmental pollution [1].

Polyurethanes are mainly obtained by reacting an isocyanate with polyesters or polyether polyols. Nevertheless, polyurethane production is highly dependent on petroleum products. Lignin bears a relatively low number of hydroxyl groups in its structure [2]. Thus, to increase the role of lignin in the formulation of polyurethanes, research has focused on how reactive its hydroxyls are as polyol precursors by modifying it rather than using native lignin directly [3]. Many studies are based on the replacement of polyols from petroleum products with those from renewable biomass resources such as wood [4], sugarcane bagasse [5], walnut shells [6], castor oil [7], linseed oil [8], soybean oil [9], palm oil [10], and corn stover [11], which could have a great impact on the polyurethane foam industry.

Lignin, due to its consistent -OH content (around 3 and 7 mmol/g), can be used as bio-based polyol to react with diisocyanate groups to form urethane bonds, which is appropriate for polyurethane cross-linking [3]. It use in this field offers many advantages such as reductions in environmental impact and cost and an increase in biodegradability. Lignin increases the hydrophobicity of PU foams by lowering its water absorption. It can be used to reinforce the PU network by increasing stability and mechanical strength [2]. Based on the nature of diisocyanate supplier, lignin as polyol leads to rigid or flexible PU foams for several applications in automotive, packaging, thermal, and phonic insulation [2,3]. However, the use of lignin in PU foam production has a negative impact on certain foam properties: it lowers the reactivity of the foaming process due to the steric hindrance of its hydroxyl groups and it makes the foam darker and results in an irregular cell structure due to high values of viscosity. Lignin can also lead to a decrease in compression strength, an increase in cell size due to the large bubbles produced, and the lowering of dimensional stability, especially when poorly dispersed [2,4,7].

Green lignin modification routes have been evaluated, such as depolymerization by hydrolysis with water [12], demethylation [13], modification [14], or simply additives [15]. One of the processes used to modify the structure of bio-based polyols from renewable sources is liquefaction [16], especially for lignin [3]. Enzymatically hydrolyzed lignin was liquefied with polyethylene glycol and glycerol by Jin et al. [17], who reported that the -OH content of liquefied products increased with the quantity of polyols [18]. The same conclusion was reached when polyethylene glycol and glycerol were used to functionalize lignin by microwave heating [3]. Zhang et al. [19] used ethylene carbonate and polyethylene glycol (PEG) as functionalization agents in oxyalkylation. Many diisocyanate compounds, such as polymeric methyl diphenyl diisocyanate (pMDI) [15,20] and toluene diisocyanate (TDI) [21], have been used based on the nature and properties of the type of foam required [20]. However, since foaming with TDI and polyester polyol is exothermic, TDI prepolymers were used instead of crude TDI.

In previous studies, Kraft and Organosolv lignins as bio-polyol precursors were almost always used [1,2,14,22]. Rarely has a non-sulfur alkali lignin been used [13]. This domain is yet to be assessed, especially using soda lignin or lignin obtained from a combination of pulping processes.

In this study, hardwood soda–ethanol lignin (SOL) extracted from sapele (*Entandrophragma cylindricum*) was used in place of a petroleum-based polyol. Liquefaction temperature was varied, lignin reacted with polyethylene glycol and glycerol, the reaction was acid-catalyzed, and three lignin-based polyols were obtained and labeled LPB1, LBP2, and LBP3 at 1.5, 2, and 2.5 h, respectively. The foams were prepared from the three bio-polyols samples reacting with a TDI prepolymer. Poly(dimethylsiloxane) as surfactant, water as blowing agent, and triethanolamine as catalyst were used, and the resulting foams (lignin-based polyurethane) were labeled LBPUF1, LBPUF2, and LBPUF3. The lignin extracted, the bio-polyols obtained, and the foams produced were characterized by several methods.

## 2. Materials and Methods

### 2.1. Materials

Sapele (*Entandrophragma cylindricum*) wood was taken at an altitude of 647 m in the eastern region of Cameroon around the Dimako Council. After air-drying the wood, it was chipped before drying for 24 days at 50 °C. After grinding (Retsch DR grinder) and sieving, 200 and 315 μm sawdust samples were obtained according to AFNOR NFB 52-001-1 standard requirements [23].

Ethanol (96%), NaOH (99%), acetone (95%), phthalic anhydride (95%), pyridine (90%) HCl (95%), and poly(dimethylsiloxane)-hydroxy were purchased (Sigma Aldrich, Burlington, MI, USA). For foam synthesis, polyethylene glycol and glycerol (Honeywell Riedel de Haen GmBH, Seelze, Germany), TDI prepolymer (%NCO = 3.3%) (Bayer, Leverkusen, Germany), and triethanolamine (Merck, Darmstadt, Germany) were purchased.

All chemicals were analytical grade and were used without further purification.

### 2.2. Methods

#### 2.2.1. Pulping and Lignin Extraction

A 0.8 L pressure-resistant steel oil bath was used for pulping. A sodium hydroxide solution of 1.872 M of alkali charge 9.95% was prepared. Then, 5 g of oven-dried sapele sawdust was loaded into the reactor containing sodium hydroxide solution at a solid/liquid mass ratio of 1:10. After an impregnation period of 15 min at ambient temperature, the temperature was increased to 180 °C. After the predetermined reaction time, the reactor was cooled. Afterwards, the pulp was filtered and the residue washed with distilled water and dried at 75 °C overnight to determine the yield. A 2% (wt.) sulfuric acid solution was then added to the previous filtrate and left for 24 h. The solution was filtered and the lignin residue was dried in an oven at 75 °C for 3 days and preserved for further analysis.

Standard methods were used to determine the chemical composition of lignin. Ash content was determined according to the TAPPI T-211 standard [24]. Determination of residual water by ethanol–benzene extraction was replaced by acetone extraction according to ASTM C 494-11 [25]. Hot water extraction followed the acetone extraction according to TAPPI T 207 cm-99 [26].

#### 2.2.2. UV Spectrophotometry of Lignin

UV spectrophotometry was carried out on a Shimadzu UV-vis spectrophotometer (Shimadzu Corporation, Tokyo, Japan) and spectra were recorded in the 300–800 nm range at 1 nm resolution. The solvent was ethylene glycol and the solid/liquid ratio was 1:5.

#### 2.2.3. Liquefaction of Lignin and Formation of Bio-Polyols

The liquefaction reagents used were glycerol and PEG-400 at a 20:80 weight-to-weight ratio, while the lignin/solvent ratio was 1:5. The reactions were all catalyzed with 1.5% sulfuric acid. Lignin (20 g) was dispersed in a flask containing 100 g of PEG-400 and glycerol and 1.5 g of sulfuric acid, and the assembly was thoroughly stirred before liquefaction. The mixture was heated in an oil bath under reflux at 140 °C for cooking times of 1.5, 2, and 2.5 h. After the reaction time, the liquefied mixture was cooled and diluted with a 1,4-dioxane–water mixture at a 4:1 *v*/*v* ratio. The residue from liquefaction was removed by filtration. The liquefaction yield was calculated using Equation (1):

(1) $$yield\left(\%\right)=\left(1-\frac{m}{m_{0}}\right)\times 100$$

where m is the mass of residue after filtration and m_0_ is the mass of lignin weighed.

**Scheme 1:**
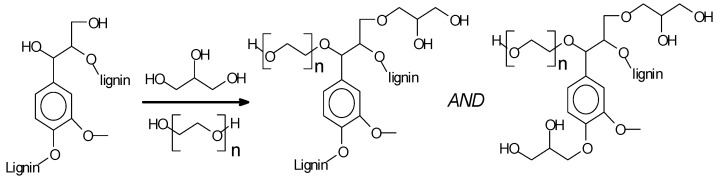
Schematic representation of two of the likely reactions between lignin and PEG/glycerol.

There are many reactions that can occur between a lignin and polyethylene glycol–glycerol mixture. Two of the most probable are represented in Scheme 1.

#### 2.2.4. Preparation of Lignin-Based Polyurethane Foams

The polyurethane foaming process requires mixing the reagents and additives in suitable proportions to ensure the foams have specific properties [27]. The NCO/OH ratio was 1.3:1 to ensure a complete process. The amounts of bio-polyols and TDI were determined based on the previous ratio, as were the NCO content of TDI and -OH groups of bio-polyols, and the formula used was:

(2) $$\frac{NCO}{OH}=\frac{m_{0}\times {\left[NCO\right]}_{0}}{m_{1}\times {\left[OH\right]}_{1}}$$

where m_0_ is the mass of TDI used (g), m_1_ the mass of bio-polyol obtained (g), [NCO]_0_ the concentration of NCO in TDI (mmol/g), and [OH]_1_ the concentration of -OH in bio-polyol (mmol/g).

The polyisocyanate used was the TDI prepolymer, the catalyst was triethanolamine, the surfactant was poly(dimethylsiloxane), and the blowing agent was water. The foams were all prepared using a one-shot method, as formulated in Table 1.

#### 2.2.5. Maldi-TOF/MS Acquisition

About 10 mg of sapele soda-oxyethylated lignin (SOL) powder was dissolved in 2 mL of acetone and mixed with another solution containing 2,5-dihydroxybenzoic acid in acetone (10 mg/mL acetone). The ion formation was enhanced by adding sodium chloride (NaCl) to the matrix. About 0.5 L of the mixture was placed on the MALDI target and introduced into the spectrometer after evaporation of the solvent [28,29].

From the MALDI-TOF spectra, the number average molecular weight (M_n_) and the weight average molecular weight (M_w_), defined in equations [26], were determined.

(3) $$M_{n}=\frac{\sum N_{i}M_{i}}{N_{i}}$$

(4) $$M_{w}=\frac{\sum N_{i}M_{i}^{2}}{\sum M_{i}}$$

where N_i_ is the number of molecules of a specific molecular weight M_i_.

#### 2.2.6. FTIR Analyses of Lignin

An ATR-FT-MIR Frontier spectrometer (Perkin-Elmer, Wellesley, MA, USA) was used to analyze approximately 2 mg of lignin powder. The spectrometer was equipped with a diamond–ZnSe crystal on which a sensor (1.8 mm) was placed on the sample to analyze it. Sufficient contact of the sample was attained by 150 N screwing force of the diamond. A total of 5 scans per extract sample were taken in the 600–4000 cm^−1^ wavelength range at 4 cm^−1^ resolution, with 32 scans per sample.

#### 2.2.7. Thermogravimetric Analyses (TGA)

A Netzsch STA 449 F3 Jupiter analyzer was used to measure the stability and thermal decomposition of polyurethane foams. Approximately 20 ± 1 mg of ground fiber sample powder was placed on the platinum stage, which was then heated to a temperature ranging from 25 to 600 °C at a rate of 20 K min^−1^ in a nitrogen atmosphere.

#### 2.2.8. Differential Scanning Calorimetry (DSC) on SOL

The glass transition temperature T_g_ of lignin was determined with a DSC 6000 (PerkinElmer, Wellesley, MA, USA) under a N_2_ gas cover. Samples (5 mg) of lignin or corresponding foams were tested at a 20 °C/min heating rate in the 23 °C to 120 °C range. The samples were then cooled to 25 °C at a rate of 20 °C/min and held at the final temperature for 10 min. The second heating cycle to determine T_g_ of the lignin samples was carried out under the same conditions, but up to 200 °C.

#### 2.2.9. Apparent Density of Foams

The apparent densities of the foams were determined according to the DIN 53479 standard [30]. The samples were first weighed in dry conditions, then weighed after dipping into water as test liquid. Density, ρ_i_ (kg/dm^3^), was calculated using the formula:

(5) $$\rho_{i}=\frac{m_{0}\;\times \;\rho_{tl}}{m_{1}-m_{0}}$$

where m_0_ is the mass of the dried sample (g), m_1_ is the volume of the wet sample, and ρ_tl_ is the density of water as test liquid (1 kg/dm^3^).

#### 2.2.10. Viscosity of Polyols

The viscosity (ƞ) was determined using a HAAKE MARS III disk rheometer (Rheology Solutions Pty Ltd., Victoria, Australia) at 25 °C in control mode in plate–plate arrangement with a diameter of 20 mm at a rotation speed of 100 cycles/min.

#### 2.2.11. Gel Permeation Chromatography (GPC)

The GPC measurements were carried out using a Knauer chromatograph equipped with a PLgel mixed-E column for the analysis of the oligomers and a refractometric detector with polystyrene as standard. Tetrahydrofuran was used as eluent at a 0.8 cm^3^/min flow rate at room temperature. The weight average molecular weight (M_w_) and the number average molecular weight (M_n_) and the polydispersity Đ of the polyols were determined.

#### 2.2.12. Study of the Foam Process

- Cream time

This is the time interval in which the mixture starts to expand. It is also called the bench time of mixing. It is measured with a stopwatch and shows periods after which a mixture turns creamy.

- Gel time

This is the time at which the mixture turns into a foam or gel due to cross-linking reactions occurring in the mixture. When the gel is formed, a thread appears between the spatula and the foam. Gel time is usually greater than cream time.

- Rise time

This is the time at which the mixture starts to rise. The reaction is complete, and the formation of foam starts. Rise time is greater than both cream time and gel time.

#### 2.2.13. Determination of the Hydroxyl Content of Polyols

This was determined using the D 4274-99 standard [31]. Initially, 0.1 mg of sample was weighed into a conical flask and 25 mL of the phthalic anhydride-pyridine reagent was added. The flask was then swirled and heated under reflux in an oil bath and the temperature maintained at (120 ± 2) °C for 1 h. The quantity of oil in the bath was kept sufficient to cover approximately half of the flask. After heating, the assembly was cooled to room temperature. The condenser was washed down with 50 mL of redistilled pyridine and the flask was removed. Phenolphthalein indicator solution (0.5 mL: 1 g of phenolphthalein in 100 mL of pyridine) was added to the flask and the solution was titrated with 0.5 N NaOH solution to a pink endpoint that persisted for at least 15 s. A blank was run in the same manner, omitting the sample [16]. The hydroxyl number (mg KOH/g) of the polyol was calculated as follows:

(6) $$hydroxyl\;number=\frac{\left(B-A\right)N}{m}\times 56.1$$

where A is the volume of NaOH required for the titration of the sample (mL), B the volume of NaOH required for the titration of the blank (mL), N the normality of the NaOH solution used, m the mass of sample used (g), and 56.1 the molar mass of potassium hydroxide (KOH) (g/mol).

Since the color at the equivalence point of the titration was pink, the solution was titrated with 0.1 N HCl until the pink disappeared, and then 1.0 mL excess was added. The new solution was back-titrated with standard 0.1 N NaOH to a pink endpoint that persisted for at least 15 s. To titrate with standard 0.1 N NaOH, a blank containing the same amount of 0.1 N HCl was added, and the reagent mixture was titrated as described previously, omitting the sample. The alkalinity correction, mg KOH/g, was calculated as follows:

(7) $$alkalinity\;correction=\frac{\left(D-C\right)N}{m}\times 56.1$$

where C is the volume of NaOH required for the titration of the sample (mL), D the volume of NaOH required for the titration of the blank (mL), N the normality of the NaOH solution used, and m the mass of sample used (g).

The hydroxyl number was corrected using the following equation.

(8) hydroxyl number corrected=hydroxyl number−alkalinity correction

The OH content was obtained by the hydroxyl number using the following equation:

(9) $$OH\;content=\frac{hydroxyl\;number}{56.1}$$

#### 2.2.14. ^13^C NMR Spectroscopy

^13^C NMR analysis was carried out in dimethyl sulfoxide-*d*_6_ (DMSO-*d*_6_) at a concentration of 50 mg/mL with TMS calibration at a relaxation time of 0.1 s, 90° pulse angle, 1.42 s acquisition time, 20 thousand scans, and 35 kHz spectral width.

#### 2.2.15. Scanning Electron Microscopy Images

The morphology of the cells in the foams was analyzed using a scanning electron microscope Hitachi TM3000 (Hitachi High-Technoloies Coporation, Tokyo, Japan). The closed cell content was measured according to the ISO 4590 standard [32].

#### 2.2.16. X-Ray Diffraction Analysis

The crystallinity of the foams was determined using the XRD method. This reveals the angle at which peaks occur and how intense they are. The test was performed using an X’Pert^3^ X-ray diffractometer (Malvern Panalytical Ltd., Malvern, UK). The diffraction angles 2θ ranged from 5° to 70° for samples in powder form. The degree of crystallinity (% Cr) was calculated using the Segal equation [33]:

(10) $$\%Cr=\left(\frac{I_{c}}{I_{c}+I_{A}}\right)\times 100$$

where I_C_ is the integrated intensity of the sharp peak of the crystalline zone and I_A_ the intensity of the broad peak of the amorphous zone.

#### 2.2.17. Water Absorption in Foams

The water absorption tests were carried out at room temperature. Samples with an approximate dimension of 15 × 15 × 15 mm^3^ were cut from each LBPUF foam and weighed. The samples were then placed in distilled water for 12 h. Afterwards, the samples were taken out, wiped with filter paper, and then weighed. The percentage weight change (% W) was determined as follows:

(11) $$\%W=\left(\frac{m_{1}-m_{0}}{m_{0}}\right)\times 100$$

where m_0_ was the initial weight of the sample and m_1_ was the final weight of the sample.

#### 2.2.18. Mechanical Tests

The compressive strength of LBPUF foam was measured according to ASTM D 1621-16 standard [34]. LBPUF foam samples of dimensions 25 × 25 × 22 mm^3^ (22 mm in the foam rise direction) were tested in triplicate using an Instron 3382 universal testing machine with a crosshead displacement rate of 2 mm/min. The experimental values were calculated using Equation (12):

(12) $$compressive\;strength=\left(\frac{d_{0}-d_{r}}{d_{0}}\right)\times 100$$

where d_0_ is the original distance between grips and d_f_ the distance between the grips at the breakpoint.

The tear test was performed according to the ASTM D3574 standard [35] test method on the Instron Universal 5565 testing machine. The rectangular samples were clamped in the jaws of the machine, ensuring that the jaws clasped the samples uniformly. The crosshead speed of grip separation was 500 × 50 mm/min. Tear strength was calculated using the equation:

(13) $$tear\;strength=\left(\frac{F}{T}\right)\times 1000$$

where F is the force (load in N) and T the thickness of the sample (mm).

The tensile strength and elongation of the LBPUFs were determined according to the UNE-EN 1798: 2008 standard [36] on an MTS Criterion 43 device. Testing was conducted at a separation rate of 500 ± 50 mm/min. Five specimens of each foam were tested, and an average value is reported. The elongation was measured using Equation (14):

(14) $$elongation=\left(\frac{d_{f}-d_{0}}{d_{0}}\right)$$

where d_0_ is the original distance between grips and d_f_ the distance between the grips at the breakpoint.

## 3. Results and Discussion

### 3.1. Characterization of Lignin

#### 3.1.1. FTIR Spectra

**Figure 1:**
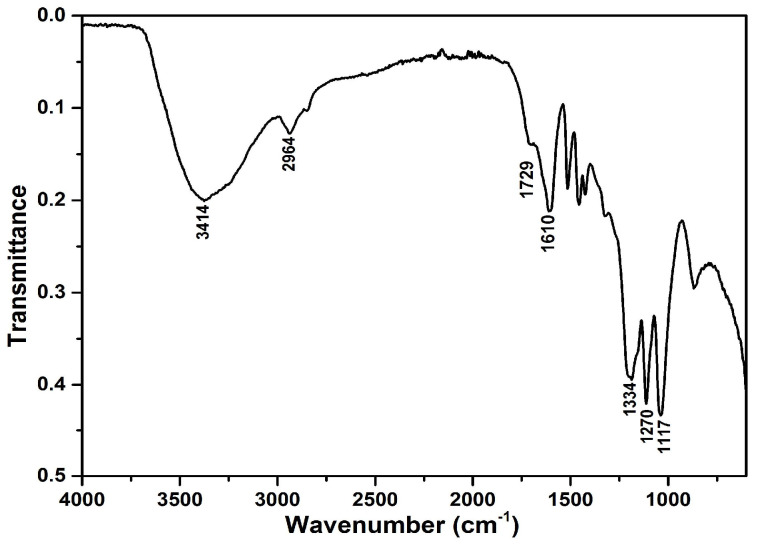
Infrared spectrum of dissolved lignin.

In Figure 1, the spectrum shows many absorption bands, the peak at 3416 cm^−1^ being the absorption peak of the hydroxyl group of an alkaline lignin. The peak at 2964 cm^−1^ is assigned to the sub-methyl group in alkaline lignin. The peak at 1729 cm^−1^ is assigned to the -C=O of ketone and carboxylic acids, while the peak at 1610 cm^−1^ is assigned to the lignin aromatic ring. The peak at 1334 cm^−1^ is assigned to the syringyl ring, the peak at 1270 cm^−1^ is assigned to the guaiacyl ring, and the peaks at 1117 cm^−1^ and 1048 cm^−1^ are assigned to -C-O of ester of an aliphatic ether and of primary alcohol [37]. The peak of the -OH band shows relatively weak intensity and hence the lignin has low hydrophilicity, meaning that for its use under hydrophilic conditions, the proportion of -OH groups must be increased.

#### 3.1.2. TG/DTG Curves of SOL

**Figure 2:**
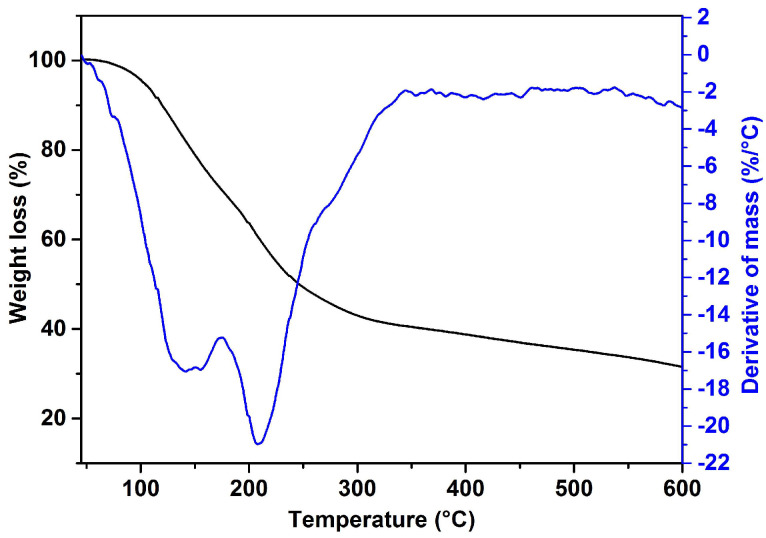
TGA and DTG thermograms of the SOL lignin sample.

In Figure 2 the TGA and DTG curves used to determine the thermal degradation of lignin are shown. The first weight loss occurred before 150 °C of solvents and water volatilization [37,38]. The weight loss that followed took place before 250 °C due to the cleavage of such bonds as α- and β-aryl-alkyl ether bonds [39]. The lignin structure was stable at high temperatures because of a great amount of branching and condensed aromatic structures.

#### 3.1.3. DSC Curve

**Figure 3:**
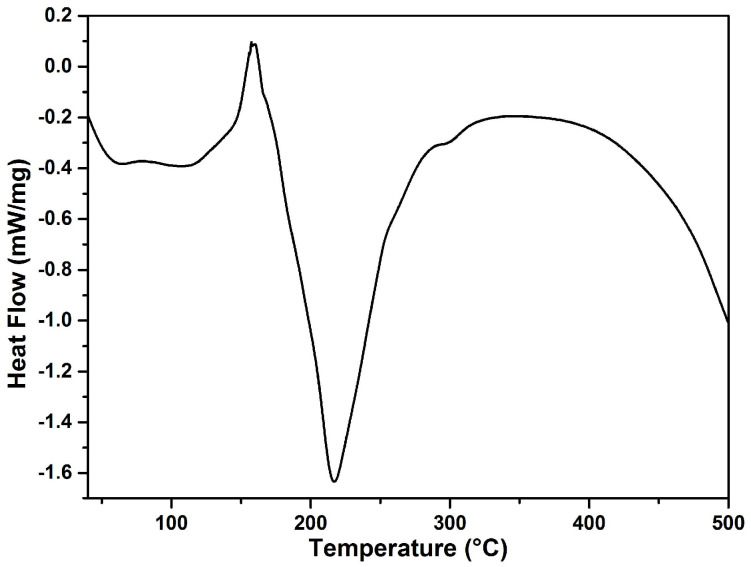
DSC curve of the SOL sample.

The thermal DSC analysis curve of lignin is shown in Figure 3. There are three peaks: two endothermic and one exothermic. The first endothermic event took place between 80 and 100 °C and can be assigned to the departure of physically absorbed water. The exothermic peak at around 150–180 °C can be assigned to the beginning of the cleavage of some less stable functional groups. The consistent endothermic peak around 200–225 °C can be assigned to the cleavage of β-O-4 ether bonds [37,38].

The glass transition temperature (T_g_) of the lignin was found to be 178 °C. This value is due to the random distribution of functional groups, hence the configurational irregularity of lignin [40].

#### 3.1.4. Other Chemical Characteristics of SOL

**Figure 4:**
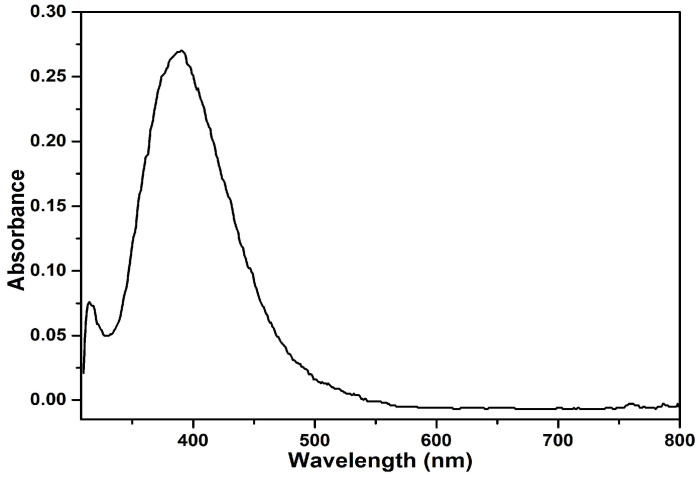
UV spectrum of the lignin sample.

Some other chemical characteristics of lignin are summarized in Table 2. The ash content of SOL was 1.12%, a value that agrees with previous studies [41]. Figure 4 shows UV-visible absorption characteristics of the lignin. The peak at 315 nm can be attributed to π → π* transitions due to the presence of an extended conjugation on the aromatic rings. The peak at 395 nm could account for the n → π* due to unconjugated chromophores on oxygen atoms of ethers, esters, and phenolic groups [42]. The number of -OH phenolic groups was small based on the UV analysis, this being due to the condensation of those groups during the pulping process [43]. This value is slightly lower than that of soda-anthraquinone lignin (6.43%) and kraft lignin (6.48%) [43]. The quantity of residual carbohydrates in SOL was also low, meaning that the soda–ethanol pulping liquor damaged more carbohydrates than the soda–anthraquinone (7.2%) and kraft (7.1%) processes. This pulping process dissolves less carbohydrate than other classical pulping processes [44].

The average molecular weights of SOL were M_w_ = 2056 g/mol and M_n_ = 885 g/mol, with a polydispersity ratio of 2.32. These values are less than those in the literature for commercial kraft lignin (M_w_ = 2792 g/mol, M_n_ = 909 g/mol and D = 3.07) [45], meaning that the soda–ethanol process only slightly damaged the lignin. 

The OH content found was 4.885 mmol/g, higher than that obtained from lignin extracted using the Organosolv process (4.26 mmol/g) [46]. This could be due to a combination of ethanol and soda as pulping liquor and the nature of the wood used.

#### 3.1.5. MALDI-TOF Spectra of Lignin

**Figure 5:**
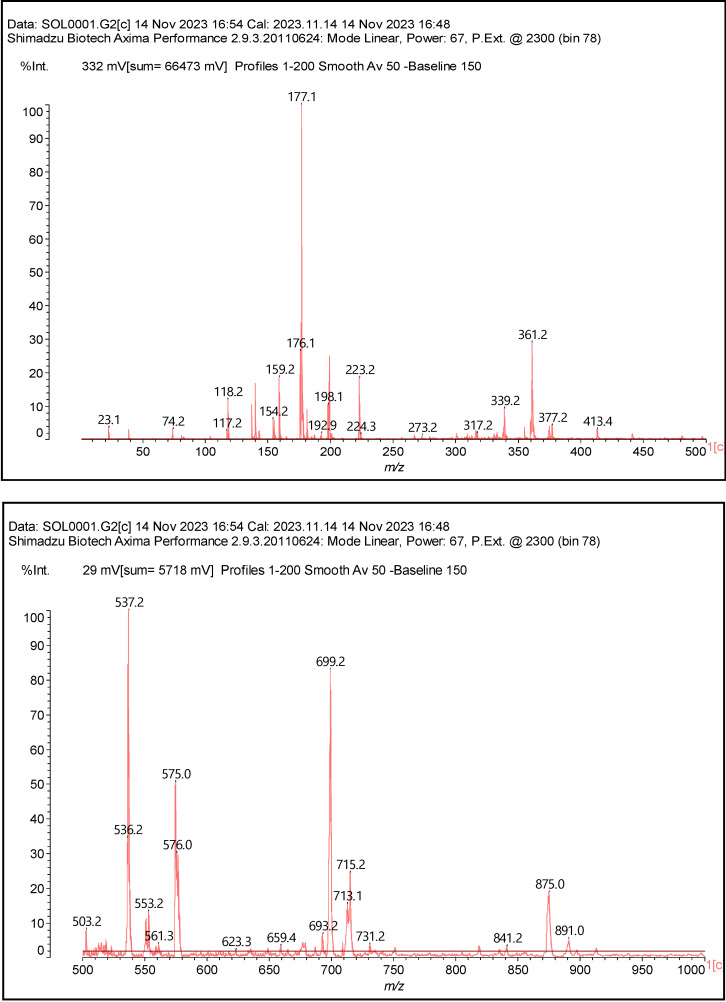

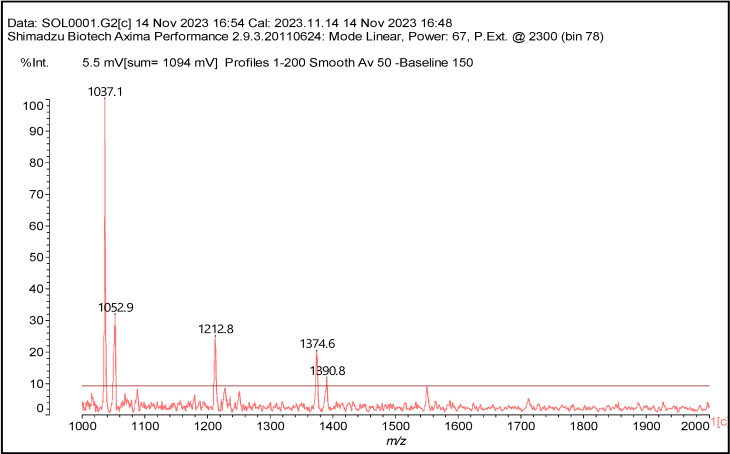
Continuous MALDI-TOF spectra of SOL.

The MALDI-TOF spectra of SOL are shown in Figure 5. These analyses were carried out in the 20–2000 Da range. Some monomers and oligomers can be noted: glucose monomer residues (177 Da), lignin units (198 and 228 Da), dilignols (317, 339, 361 and 377 Da), trilignols (561 and 575 Da), tetralignols (699 and 715 Da), pentalignols (875 and 890 Da), hexalignols (1037 Da), and heptalignols (1212 Da). Lignin units and their alcohol polymers predominate in the lignin sample. The presence of Na^+^ in the matrix throughout the analysis led to an increase in molecular weight by 23 Da of some monomers (198, 223, 339 and 1037 Da), while others were reduced (177, 198, 317, 361, 377, 561, 575, 699, 715, 890 and 1212 Da) [47]. Other monomers gained or lost hydrogen (223, 317, 339, 361 and 561 Da), or lost -OH groups (875 Da). This shows that the SOL sample had many -OH groups that were almost all relatively hindered [48,49].

### 3.2. Characteristics of Lignin-Based Bio-Polyols (LBPs)

#### 3.2.1. Liquefaction Yield, Viscosity, and Hydroxyl Content of the Bio-Polyols

The values obtained for the liquefaction yield, viscosity and hydroxyl content as a function of cooking time of the bio-polyols are summarized in Table 3. The liquefaction yield increased with cooking time, meaning that the longer the treatment, the better the functionalization of lignin [14,50]. The LBP liquefaction percentage yield was comparable to that obtained by Jin et al. [17], who reported that under conventional heating, the liquefaction yield was 98.4%.

The viscosity of polyols is a critical factor that must be taken into account in the preparation of PU foam: high viscosity values can be burdensome when mixing foam ingredients and for the distribution of cells formed by CO_2_ departure [22]. When cooking time increased, the values of viscosity of the bio-polyols also increased slightly from 11.3 to 13.6 cP. This variation could be assigned to a greater degradation of lignin in the bio-polyols [3].

The hydroxyl content of the bio-polyols increased from 6.973 to 7.637 mmol/g. This increase is slight between 120 and 150 min of cooking time. This could be due to a complete reaction of hydroxyl groups in lignin leading to a marked reduction in active sites, making their participation in subsequent reactions difficult [51].

#### 3.2.2. FTIR Spectra of the Bio-Polyols

**Figure 6:**
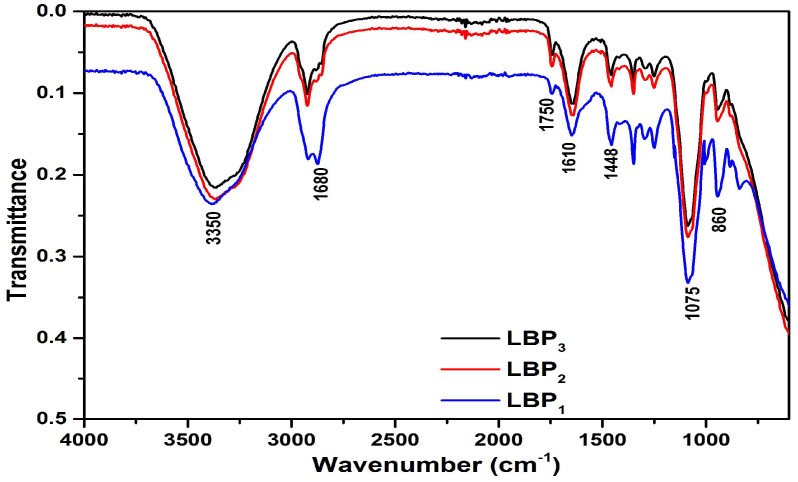
FTIR spectra of bio-polyols.

The FTIR spectra of the bio-polyols (liquefied products) are shown in Figure 6. The bands around 3350 cm^−1^ are assigned to hydroxyl stretching, and the band at 860 cm^−1^ is assigned to the CHO adsorption peak, which indicates that glycerol and polyethylene glycol (PEG) may have undergone isomerization to form an aldehyde structure. The band at 1750 cm^−1^ is assigned to C=O stretching and that at 1075 cm^−1^ to C-O stretching [3]. Both the -CO and -OH groups presented higher intensity than that observed in the SOL FTIR lignin spectra, meaning that the proportion of -OH groups increased during the liquefaction or bio-polyol preparation [51]. The bands around 1585 cm^−1^ and 1611 cm^−1^ are assigned to the aromatic rings of lignin, but they are not easily observed. This could be due to the breakdown of the bonds, which appeared during the coupling of aromatic rings [17]. These results corroborate those of the corresponding molecular weights. The PEG and glycerol distortion in symmetrical mode of the -CH_2_- groups is represented by the 1448 cm^−1^ band. The band of the carbon to oxygen bond carrying the lignin’s -OHs underwent a shift from 1220 to 1245 cm^−1^, indicating a reaction of the bio-polyol solvents with the lignin phenylpropane units -OHs [17].

3.2.3. ^13^C NMR Spectra of Bio-Polyols

**Figure 7:**
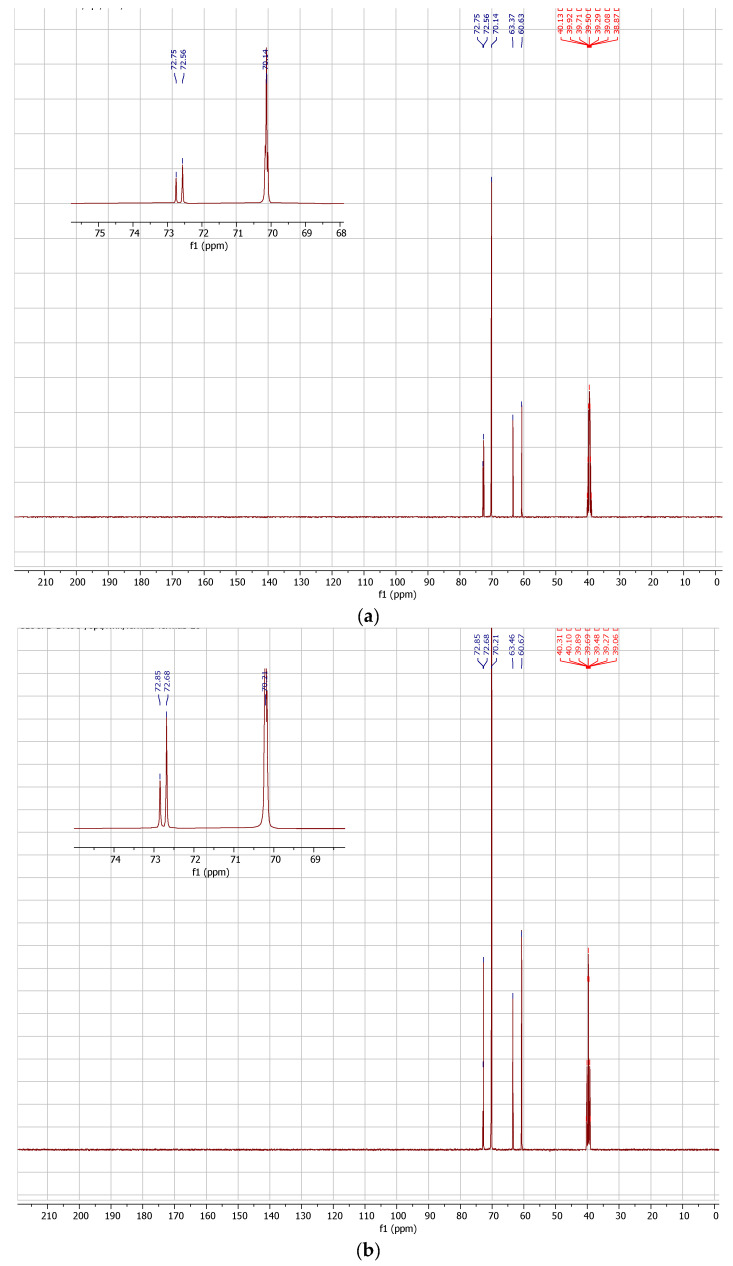

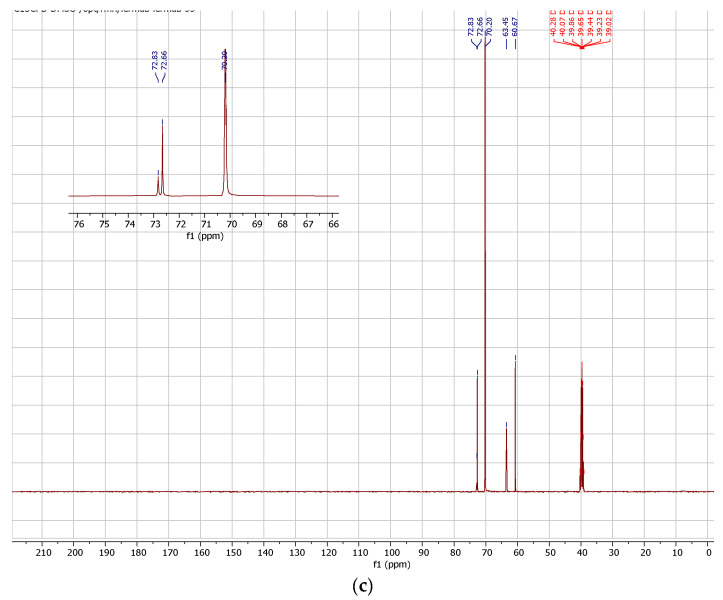
^13^C NMR spectra of bio-polyols: (**a**) LBP1, (**b**) LBP2 and (**c**) LBP3.

The ^13^C NMR spectra of bio-polyols are shown in Figure 7. The peaks at 70.21 and 60.67 ppm are assigned to secondary and primary carbons of PEG, respectively. The peaks at 72.85 and 63.46 ppm are assigned to secondary and primary carbons of glycerol, respectively [50]. In the 140 ppm area, no visible peak is observed, meaning that there is no consistent aromatic carbon of ether. This can be ascribed to lignin’s phenolic -OHs [3]. These results corroborate the FTIR analyses.

#### 3.2.4. Molecular Weights of the Bio-Polyols

Table 4 presents the values of weight average (M_w_) and number average (M_n_) molecular weights, as well as the M_w_/M_n_ polydispersity ratio of the bio-polyols.

Compared to lignin, the molecular weights of bio-polyols fell drastically. This could be due to the cleavage of lignin through the incorporation of a great amount of glycerol and PEG [3]. Conversely, the molecular weights of bio-polyols increased with cooking time. These results could be due to a condensed structure obtained by the incorporation of glycerol and PEG into the lignin structure [45]. It could also be due to a self-polymerization reaction that could have occurred during lignin liquefaction, leading to its degradation to fragments of smaller molecular weight. The ether bonds in bio-polyols were hence formed from the reaction of -OH groups with PEG and the fragments could have finally recondensed to form the residue.

### 3.3. Study of the Foaming Process

#### 3.3.1. Foaming Images

**Figure 8:**
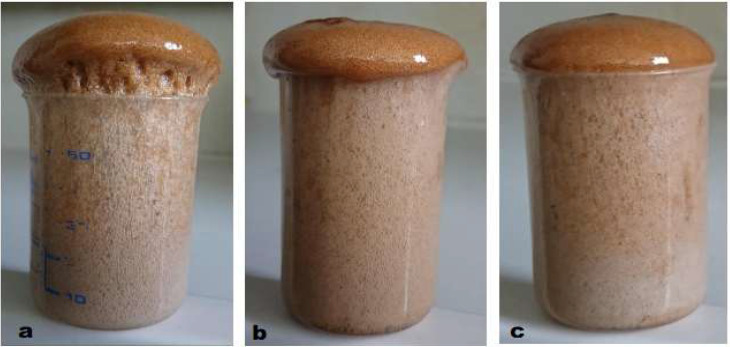
Photographs of foams: (**a**) LBPUF1, (**b**) LBPUF2, (**c**) LBPUF3.

Photographs of the foams are shown in Figure 8. The brown color of the samples is due to the presence of lignin in the polyols. The expansion of the foams is related to the -OH content in bio-polyols: it increases with the height of the foams produced, and the expansion of the foams is mainly due to the amount of CO_2_ liberated during the foaming process [15].

#### 3.3.2. Kinetics of the Foaming Process

**Figure 9:**
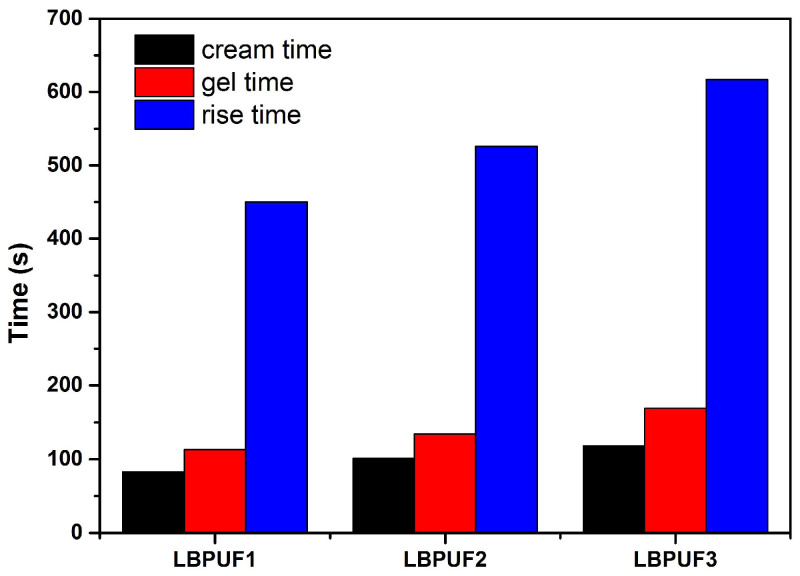
Foaming characteristic times of lignin-based polyurethane foams.

The values of cream time, gel time, and rise time of the foams are given in Figure 9. The foaming rate increased with the number of -OH groups of the bio-polyols used. This is due to the active interaction and the availability of -OH groups on the TDI prepolymer, which leads to a faster reaction. While the proportion of -OH groups on the polyols increased, a decrease in the proportion of residual NCO isocyanate groups capable of reacting with water also decreased, thereby also diminishing CO_2_ production. Thus, bubble cell formation by foaming was hindered by an increase in viscosity of the medium. The decrease noted in cream times could be due to the increase in viscosity of the bio-polyols [6].

### 3.4. Characteristics of Lignin-Based Polyurethane Foams (LBPUFs)

#### 3.4.1. Surface Morphology and Density of Foams

**Figure 10:**
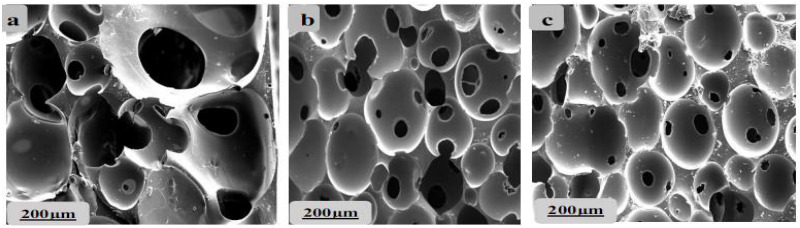
SEM micrographs of (**a**) LBPUF1, (**b**) LBPUF2, and (**c**) LBPUF3.

The scanning electron microscopy micrographs in Figure 10 show the surface morphologies of lignin-based polyurethane foams (LBPUFs). The cell structure of the foams is a significant parameter that explains the properties of the foams [52,53]. The dark color of the LBPUFs indicates their suitability for use in building applications [52]. The cell surfaces of the LBPUFs were mostly smooth, with some irregularities: closed and open gaps were observed, and the cell morphology of the LBPUFs was very similar to that of those seen in literature [52]. The LBPUF1 sample presented oval and larger cells. The sizes were almost the same. For LBPUF2 and LBPUF3, the cells were regular. The higher the preparation temperature of bio-polyols, the smaller the cells. The same was true for agglomeration in foams with an increase in preparation temperature of the bio-polyols. The increase in preparation temperature of bio-polyols caused the cell shape to become less regular [54].

The mean cell diameter and the density of the foams are shown in Table 5. The sample produced with polyol obtained at 1.5 h showed the highest density. This can be explained by the shrinkage after the foaming due to the polyol, water, and some unconverted PEG or glycerol evaporating [50,55]. The increase in temperature observed could be due to a decrease in water content of the polyols [19]. An increase in liquefaction temperature could have caused a decrease in the density of the foams prepared. This is because the higher the liquefaction temperature, the higher the proportion of -OH groups of the corresponding polyols, which consume an equivalent quantity of TDI prepolymer, hence the lower the residual TDI prepolymer, the lower the amount of CO_2_ produced during the foaming process [19].

#### 3.4.2. FTIR Spectra of Lignin-Based Polyurethane Foams

**Figure 11:**
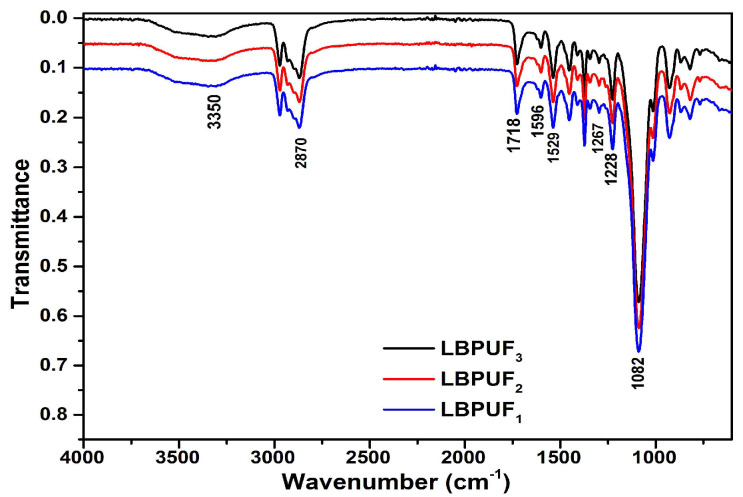
FTIR spectra of lignin-based polyurethane foams.

Figure 11 shows the infrared spectra of bio-polyols (liquefied products). The isocyanate groups of the TDI are meant to react with the hydroxyl groups at the surface of the lignin. The bands around 3350 cm^−1^ can be assigned to N-H stretching vibration [56] and the bands around 1718 cm^−1^ to C=O vibration, which is the main carbon in the polyurethane foam structure [9]. The bands around 1596 cm^−1^ are assigned to the aromatic ring of TDI, the bands around 1529 cm^−1^ are related to the N-H bending vibration, the band at 1228 cm^−1^ is related to C-N stretching, while the bands around 1082 cm^−1^ are assigned to C-O stretching [4]. Only one guaiacyl unit characteristic band is seen around 1267 cm^−1^, but its intensity is very weak [51]. This corroborates the results of FTIR and ^13^C NMR of bio-polyols. On all the spectra, no band was observed around 2270 cm^−1^, meaning that all NCO groups of the TDI prepolymer reacted with -OH of the bio-polyols.

#### 3.4.3. TG/DTG Curves of Lignin-Based Polyurethane Foams

**Figure 12:**
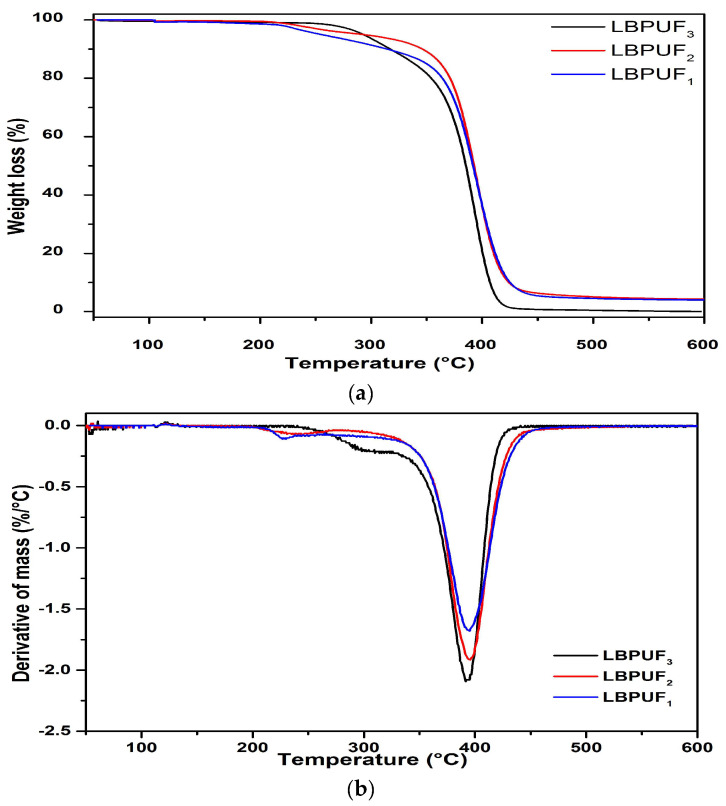
(**a**) TGA and (**b**) DTG thermograms of lignin-based polyurethane foams.

The thermal stability of the foams was studied using TG and DTG, as shown on Figure 12. All the foam samples were degraded at a temperature of 200–600 °C in two steps. A slight weight loss was observed before 270 °C, which could be due to the degradation of some small unreacted molecules, such as bio-polyols [56]. The second degradation occurred between 270 and 500 °C, which could be due to the decomposition of urethane linkages in foams [15]. When the thermal decomposition temperature increases, the number on polar groups increase and the hydrogen bonds are stronger. In the same way, the intermolecular strength increases, and thus the thermal stability of LBPUFs is improved [51].

The thermogravimetric parameters, including the char residue at 600 °C, are given in Table 6. The increase in the proportion of -OH groups of the polyols decreased T_0_ and increased T_max_ for every foam sample. The char residue was greatest for the first two foams, but lowest for the last.

#### 3.4.4. XRD Patterns of Lignin-Based Polyurethane Foams

**Figure 13:**
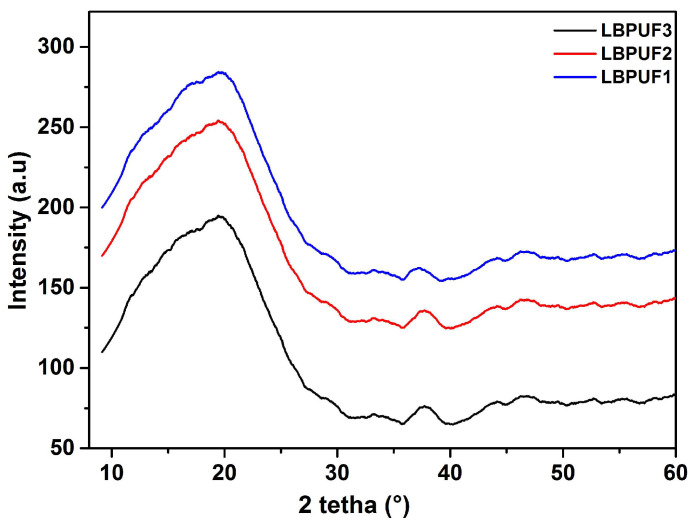
XRD patterns of lignin-based polyurethane foams.

The XRD patterns of LBPUFs are shown in Figure 13. For all the samples, intense peaks around 19.3° represent the semicrystalline parts of the foams. This is due to the formation of the cross-linked structure involving TDI, bio-polyols, and surfactant. Such bond formation restricted the molecular mobility to form a crystal structure. The crystallinities of the foams were 30.24%, 29.88%, and 27.18% for LBPUF1, LBPUF2 and LBPUF3, respectively. This could be due to the proportion of -OH groups in the bio-polyols: the higher the proportion, the more crystalline the foam, and hence the greater the porosity [39].

#### 3.4.5. Mechanical Properties of LBPUFs

**Figure 14:**
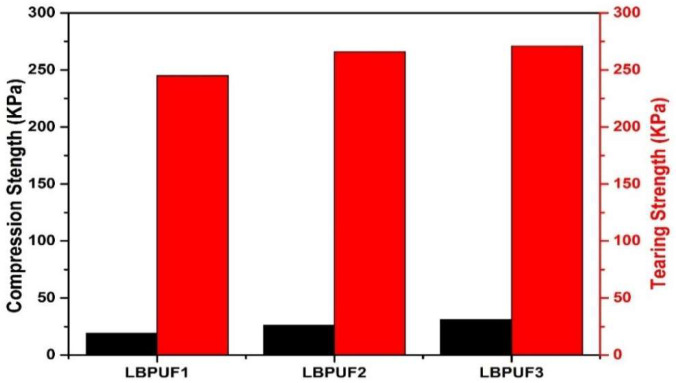
Effect of liquefaction temperature on compressive strength and tearing strength.

Figure 14 presents the effect of liquefaction temperature on foam compression and tearing strengths. The compression strength of the different LBPUFs increased from 24 to 29 kPa with the liquefaction temperature. There is a strong correlation between the compression strength of the foam and the proportion of -OH groups in the bio-polyols, with higher proportions contributing to more compact foams and denser structures. An increase in foam density has a significant effect on the compression strength [19]. The tearing strengths of all LBPUFs are within the range 248–271 kPa. The strength increases with the liquefaction temperature, meaning that the tearing strength increases when the cell size decreases, and hence the amount of CO_2_ molecules produced during the foaming. The tearing strengths of the foams are between 200 and 1000 kPa, meaning that LBPUFs can be used in automotive applications and floor carpeting [46].

**Figure 15:**
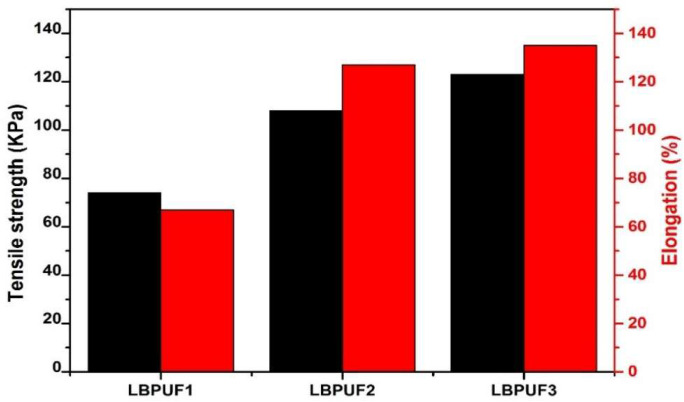
Effect of liquefaction temperature on tensile strength and elongation.

The foam elongation and tensile strength are presented in Figure 15, showing the effect of the liquefaction temperature. The foam tensile strength ranged in the 73–124 kPa interval, while the elongation also increased (from 56% to 135%), both increasing with the liquefaction temperature. This is due to the aromatic rings in bio-polyols and their high -OH proportion, which leads to a higher degree of cross-linking [57]. The presence of aromatic rings in polyols lowers the elasticity and flexibility of LBPUFs, hence improving the mechanical properties of the foams.

#### 3.4.6. Water Uptake of LBPUFs

**Figure 16:**
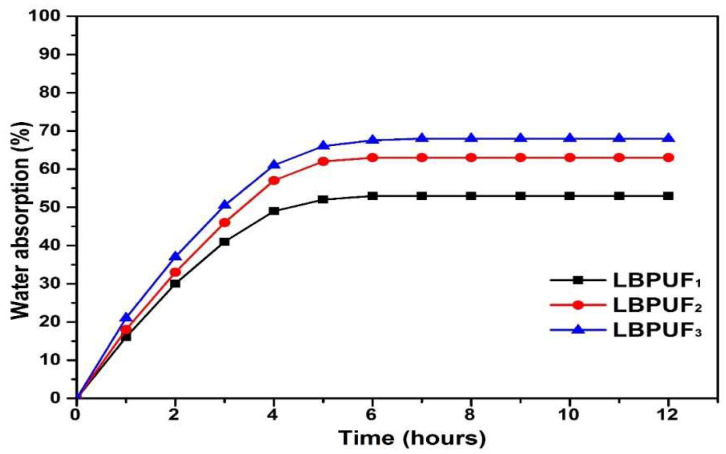
Water absorption of lignin-based polyurethane foams.

The results of water absorption tests are presented in Figure 16. These measure the amount of water gain after 12 h of contact. These results show that the more the bio-polyol is -OH- rich, the more the foam will absorb water. For all the samples, in the initial four hours, water uptake increased significantly. In the following 2 h, the water absorption reached equilibrium. This could be due to the cell sizes, which also decreased with the proportion of -OH groups [9]. Open pores absorb more water and thus improve the absorption ability of the foams [58].

## 4. Conclusions

The SOL extracted from sapele was thermally stable with a low glass transition temperature (57.4 °C). This lignin had good average molecular weights, good proportions of -OH groups, and low amounts of ash (1.12%), residual carbohydrates (1.83%), and free phenolic -OHs (3.64%). The MALDI-TOF spectra identified fragments characteristic of lignin. The chain extension of the alkaline lignin through liquefaction at different temperatures produced bio-polyol intermediates that exhibited good properties, such as viscosity, close to 99% liquefaction, and good average molecular weights and polydispersity. The bio-polyols had more -OH groups than SOL based on the -OH content, which increased after liquefaction. This was also confirmed by FTIR. These products were mixed with a TDI prepolymer and other additives, with an NCO/OH ratio of 1.3. The increase in liquefaction temperature led to an increase in number of -OH groups of bio-polyols and a decrease in cell size and density based on the amount NCO groups of TDI prepolymer that reacted with water. This trend was also responsible for the mechanical properties observed. The foams exhibited good water absorption with low crystallinity and good thermal stability (around 400 °C). The FTIR spectra showed bands characteristic of polyurethane foams: all the NCO groups appeared to have been consumed, since no residual NCO band was noted. Based on these properties, especially the presence of some aromatic rings in the bio-polyols, the foams produced can be useful in automotive applications and for floor carpets.

## Figures and Tables

**Table 1:** Lignin-bio-based polyurethane foams formulation.

Component	Part by Mass (%)
Bio-polyol	100
TDI prepolymer	2225
Surfactant	2.5
Blowing agent	1.5
Catalyst	2

**Table 2:** Other chemical characteristics of SOL.

Ash Content (%)	Carbohydrate (%)	-OH Phenolic (%)	M_w_ (g/mol)	M_n_ (g/mol)	M_w_/M_n_	-OH Content (mmol/g)
1.12	1.83	3.64	2056	885	2.32	4.885

**Table 3:** Liquefaction yields, viscosities, and hydroxyl number of bio-polyols.

Bio-Polyols	Cooking Time (min)	Liquefaction Yield (%)	Viscosity (cP)	Hydroxyl Number (mmol/g)
LBP1	90	94.27	11.3	6.973
LBP2	120	96.38	12.9	7.446
LBP3	150	98.51	13.6	7.637

**Table 4:** Average molecular weights of SOL and bio-polyols.

Samples	Lignin	LBP1	LBP2	LBP3
M_w_ (g/mol)	2056	687	894	1106
M_n_ (g/mol)	885	453	471	490
M_w_/M_n_ (Đ)	2.32	1.52	1.90	2.25

**Table 5:** Density and mean cell diameter of LBPUFs.

Sample	Cell Diameter (μm)	Density (kg/m^3^)
LBPUF1	605 ± 6	39 ± 3
LBPUF2	546 ± 2	24 ± 2
LBPUF3	482 ± 5	18 ± 3

**Table 6:** Thermogravimetric parameters of lignin and LBPUFs.

Sample	T_0_ (°C)	T_max_ (°C)	T_50%_ (°C)	Char Residue (%)
lignin	184	216	244	31.7
LBPUF1	347	389	398	35.6
LBPUF2	345	394	405	36.1
LBPUF3	345	395	407	3.9

## Data Availability

All the data that support the findings of this study are available from the corresponding author on reasonable request.
